# Use of Artificial Intelligence in the Search for New Information Through Routine Laboratory Tests: Systematic Review

**DOI:** 10.2196/40473

**Published:** 2022-12-23

**Authors:** Glauco Cardozo, Salvador Francisco Tirloni, Antônio Renato Pereira Moro, Jefferson Luiz Brum Marques

**Affiliations:** 1 Federal Institute of Santa Catarina Florianópolis Brazil; 2 Federal University of Santa Catarina Florianopolis Brazil

**Keywords:** review, laboratory tests, machine learning, prediction, diagnosis, COVID-19

## Abstract

**Background:**

In recent decades, the use of artificial intelligence has been widely explored in health care. Similarly, the amount of data generated in the most varied medical processes has practically doubled every year, requiring new methods of analysis and treatment of these data. Mainly aimed at aiding in the diagnosis and prevention of diseases, this precision medicine has shown great potential in different medical disciplines. Laboratory tests, for example, almost always present their results separately as individual values. However, physicians need to analyze a set of results to propose a supposed diagnosis, which leads us to think that sets of laboratory tests may contain more information than those presented separately for each result. In this way, the processes of medical laboratories can be strongly affected by these techniques.

**Objective:**

In this sense, we sought to identify scientific research that used laboratory tests and machine learning techniques to predict hidden information and diagnose diseases.

**Methods:**

The methodology adopted used the population, intervention, comparison, and outcomes principle, searching the main engineering and health sciences databases. The search terms were defined based on the list of terms used in the Medical Subject Heading database. Data from this study were presented descriptively and followed the PRISMA (Preferred Reporting Items for Systematic Reviews and Meta-Analyses; 2020) statement flow diagram and the National Institutes of Health tool for quality assessment of articles. During the analysis, the inclusion and exclusion criteria were independently applied by 2 authors, with a third author being consulted in cases of disagreement.

**Results:**

Following the defined requirements, 40 studies presenting good quality in the analysis process were selected and evaluated. We found that, in recent years, there has been a significant increase in the number of works that have used this methodology, mainly because of COVID-19. In general, the studies used machine learning classification models to predict new information, and the most used parameters were data from routine laboratory tests such as the complete blood count.

**Conclusions:**

Finally, we conclude that laboratory tests, together with machine learning techniques, can predict new tests, thus helping the search for new diagnoses. This process has proved to be advantageous and innovative for medical laboratories. It is making it possible to discover hidden information and propose additional tests, reducing the number of false negatives and helping in the early discovery of unknown diseases.

## Introduction

### Background

The large amount of data generated in the last decades has become a great challenge, demanding new forms of analysis and processing of complex and unstructured data, known until now as data mining [1]. The health care domain has great prominence in applying data mining, supporting infection control, epidemiological analysis, treatment and diagnosis of diseases, hospital management, home care, public health administration, and disease management [2]. This predictive analysis is strongly linked to the evolution of artificial intelligence (AI) techniques such as machine learning (ML). These algorithms, able to learn interactively from data, allow systems based on computational intelligence to find information that was initially unknown [3].

Currently, prediction systems [4] and decision-making support have been using web-based medical records and clinical data, analyzing the history of patients to propose models to identify high-risk situations as well as false positives [5]. This precision medicine (in silico) based on electronic health records has gained strength given the possibility of more accessible and efficient treatments aimed at the particular characteristics of each individual. In this sense, Wong et al [6] proposed using ML to structure and organize stored data and for mining and aiding in diagnosis. Similarly, Roy et al [7] used electronic health record data to predict laboratory test results in a pretest.

These works motivated us to study the potential of the use of AI, especially ML techniques, in the area of health.

According to Peek et al [8], in recent decades, there has been a major shift from knowledge-based to data-oriented methods. Analyzing 30 years of publications from the International Conference on Artificial Intelligence in Medicine, an increase in the use of data mining and ML techniques was observed.

In recent years, other reviews have been published presenting the growth and potential of the use of ML methods in the health area. In their review, Rashidi et al [9] addressed the multidisciplinary aspect of this scenario and presented the potential of using ML techniques in data processing in the health area comparing the different methods.

Similarly, Ahmed et al [10] discussed aspects of precision medicine in their review, presenting works with different approaches to the use of ML in addition to discussing ethical aspects and the management of health resources.

However, the work by Houfani et al [11] focused on the prediction of diagnoses, presenting an overview of the methods applied in the prediction of diseases.

In their work, Ma et al [12] present aspects of real-world big data studies with a focus on laboratory medicine. In their review, Ma et al [12] highlighted the lack of standardization in clinical laboratories and the difficulty in using data in real time, mainly because of unstructured and unreliable data. However, the potential is emphasized in the use of laboratory data together with aspects such as the establishment of the reference range, quality control based on patient data, analysis of factors that affect analyte test results, establishment of diagnostic and prognostic models, epidemiological investigation, laboratory management, and data mining. All of this is aimed at helping traditional clinical laboratories develop into smart clinical laboratories.

In contrast to the studies presented, this study aimed to analyze studies that used data from laboratory tests together with AI techniques to predict new results.

### Study Questions

Clinical laboratories display most test results as individual numerical values. However, the results of these tests, viewed in isolation, are usually of limited significance for reaching a diagnosis.

In their study of ferritin, Luo et al [5] found that laboratory tests often contain redundant information.

Similarly, Gunčar et al [13] found that ML models can predict hematological diseases using only blood tests. In their study, Gunčar et al [13] stated that laboratory tests have more information than health professionals commonly consider.

Demirci et al [14] and Rosenbaum and Baron [15] also used ML techniques to identify possible errors in the clinical process of performing laboratory tests. In both studies, the authors obtained satisfactory results, demonstrating the ability of computational models based on ML to assist in analyzing laboratory tests. Similarly, Baron et al [16] used an algorithm to generate a decision tree capable of identifying tests with possible problems arising from the preanalytical process during the execution of laboratory tests.

The presentation of these works makes us reflect on how much information can be present in a set of laboratory test data and the potential for the exploration and use of such data. Thus, our objective was to identify scientific studies that used laboratory tests and ML models to predict results.

This study had the following specific research questions: (1) Is it possible to predict specific examinations from other examinations? (2) Which examinations are typically used as input data to predict other results? and (3) What methods are used to predict these tests?

## Methods

### Search Strategy

Searches were conducted in 7 electronic databases in international journals in the areas of engineering and health sciences—Compendex (Engineering Village), EBSCO (MEDLINE complete), IEEE Xplore, PubMed (MEDLINE), ScienceDirect, Scopus, and Web of Science—in the English language for publications from April 2011 to February 2022. Additional records were further identified during the screening phase of this research by analyzing the references of the eligible articles included.

The population, intervention, comparison, and outcome principles were used to group the search terms. As this study addressed laboratory tests, 3 principal search terms were considered, and 2 Boolean operators were used (OR and AND): population (“Clinical Laboratory Test” OR “Laboratory Diagnosis” OR “Blood Count, Complete” OR “Routine Diagnostic Test”) AND intervention (“Machine Learning”) AND outcomes (“Clinical Decision-Making” OR “Computer-Assisted Diagnosis” OR “Predictive Value of Tests”).

The search terms were defined based on the list of terms used in the Medical Subject Heading database [17]. The studies were collected from the databases from April 2, 2021, to April 10, 2021; the roots of the words and all the variants of the terms were searched (singular or plural, past tense, gerund, comparative adjective, and superlative, when possible). The following filters were used for the area of activity: medicine, engineering (industrial, biomedical, electrical, manufacturing, and mechanics), robotics, health professions, and multidisciplinary according to the availability in the database.

The following study characteristics were extracted and described: authors’ names, year of publication, title, description, data set, features, methods, and main results. The data of this study were presented descriptively and followed the PRISMA (Preferred Reporting Items for Systematic Reviews and Meta-Analyses) statement flow diagram [18] and the National Institutes of Health (NIH) Quality Assessment Tool for Observational Cohort and Cross-Sectional Studies [19].

### Inclusion and Exclusion Criteria

The criteria for inclusion and exclusion of studies are outlined in Textbox 1.

The search results were exported to the web-based Mendeley software (Elsevier), where duplicates or triplicates were removed, and full texts were extracted after analyzing the possible eligibility of the articles.

**Study inclusion and exclusion criteria.**

**Inclusion criteria**

- Use of laboratory tests
- Use of machine learning techniques
- Written in English
- Full-text articles published in specialized journals

**Exclusion criteria**

- No use of laboratory tests
- Not seeking to predict new results

### Study Analysis

Regarding the eligibility of the studies, the review process involved an analysis of the title keywords and reading of the abstracts by 2 reviewers independently (the first 2 authors of this paper). When in doubt about eligibility, the full text was reviewed. In cases of disagreement between the 2 reviewers, a decision was made by consensus or a third investigator provided an additional review, and the decision was made by arbitration.

### Methodological Quality Assessment of the Studies

Regardless of the inclusion and exclusion criteria, which were directly related to the objective of the study, an analysis of the quality of the selected articles was also conducted.

The quality of the eligible studies was assessed using tools proposed by the NIH of the United States [19]. This study included the cross-sectional study assessment tool (with 14 criteria). The NIH website [19] provides tools and guidelines for assessing the quality of each type of study, containing explanatory information about each item that should be assessed in the study: (1) Was the research question or objective in this study clearly stated? (2) Was the study population clearly specified and defined? (3) Was the participation rate of eligible persons at least 50%? (4) Were all the participants selected or recruited from the same or similar populations (including the same period)? Were inclusion and exclusion criteria for being in the study prespecified and applied uniformly to all participants? (5) Was a sample size justification, power description, or variance and effect estimates provided? (6) For the analyses in this study, were the exposures of interest measured before the outcomes were measured? (7) Was the time frame sufficient so that one could reasonably expect to see an association between exposure and outcome if it existed? (8) For exposures that can vary in amount or level, did the study examine different levels of exposure as related to the outcome (eg, categories of exposure or exposure measured as a continuous variable)? (9) Were the exposure measures (independent variables) clearly defined, valid, reliable, and implemented consistently across all study participants? (10) Was the exposure assessed more than once over time? (11) Were the outcome measures (dependent variables) clearly defined, valid, reliable, and implemented consistently across all study participants? (12) Were the outcome assessors blinded to the exposure status of participants? (13) Was loss to follow-up after baseline 20% or less? and (14) Were key potential confounding variables measured and adjusted statistically for their impact on the relationship between exposure and outcome?

The rating quality was classified as good, fair, or bad, allowing for the general analysis of the evaluators considering all items [19]. Each item in the assessment tool received an “✓” rating when the study was performed, a negative (“–”) when not performed, and other options (cannot be determined, not applicable, and not reported).

According to Wong et al [20], observational studies with a classification of ≥67% of positive items indicated good quality, 34% to 66% of positive verifications indicated regular quality, and ≤33% indicated low quality.

## Results

**Figure 1:**
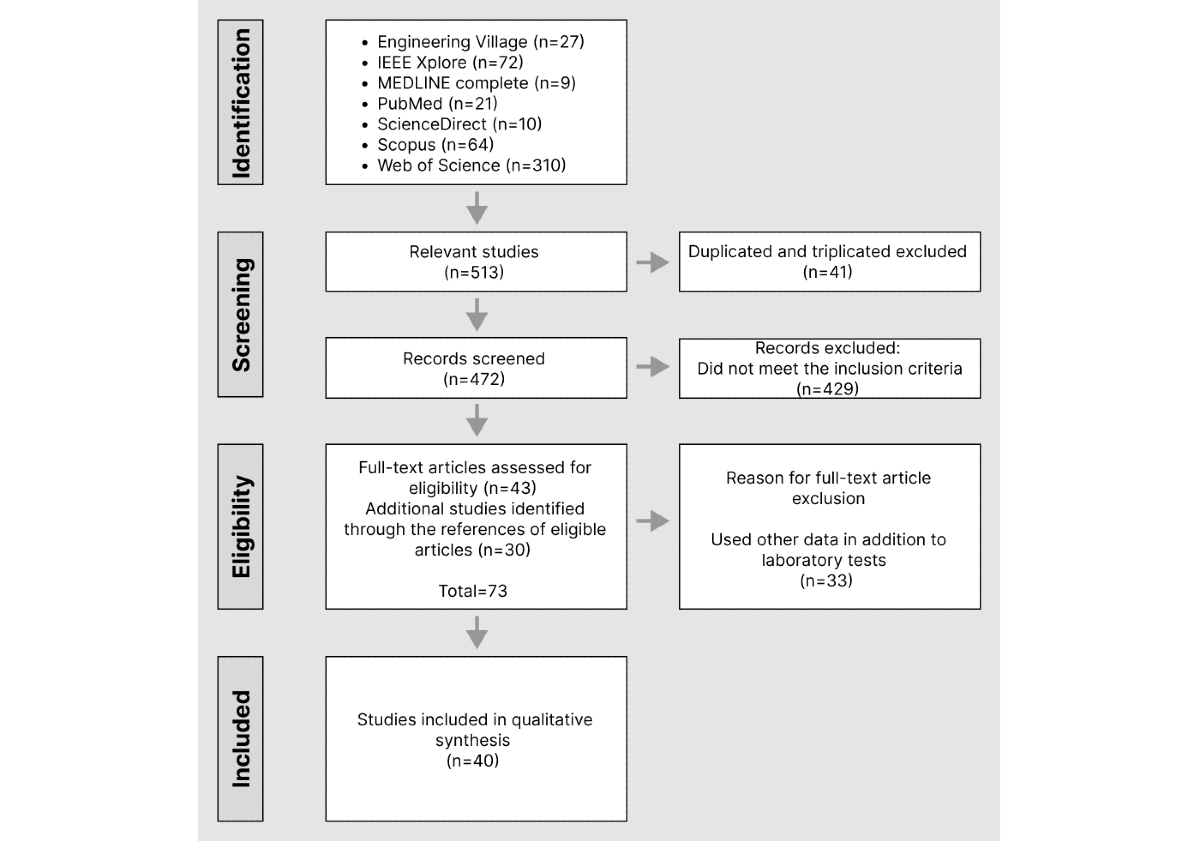
PRISMA (Preferred Reporting Items for Systematic Reviews and Meta-Analyses) flow diagram of study screening and selection.

The search results included 513 potentially eligible studies. First, 8% (41/513) of duplicated or triplicated articles were excluded, and of the 472 remaining articles, 43 (9.1%) were considered eligible based on the review of titles, keywords, and abstracts. Additional studies (n=30) were included after searching the references and citations of the eligible articles, totaling 73 full texts for evaluation. After reviewing these 73 studies, 33 (45%) were ineligible, ending the process with 40 (55%) studies for quality assessment (Figure 1).

Table 1 presents the assessment of the methodological quality of the studies. The articles are organized by author and year, by framing of the questions, and by the average points obtained through this analysis performed by the authors of this paper.

Table 2 shows the description of the studies included in this review. It is organized by author and year, title, description, data set, features, methods, and main results.

**Table 1:** Assessment of the methodological quality of the studies^a^.

Author, year	Quality assessment tool items															Total assessment tool items, n (%)
	1	2	3	4	5	6	7	8	9	10	11	12	13	14		
Richardson and Lidbury [21], 2013	✓	✓	✓	✓	✓	✓	✓	✓	✓	N/A^b^	✓	✓	✓	✓	13 (93)	
Waljee et al [22], 2013	✓	✓	✓	✓	✓	✓	✓	✓	CD^c^	N/A	CD	✓	✓	✓	11 (79)	
Kinar et al [23], 2016	✓	✓	✓	CD	✓	✓	✓	CD	✓	✓	✓	✓	✓	✓	12 (86)	
Luo et al [5], 2016	✓	✓	✓	✓	✓	✓	✓	✓	✓	N/A	✓	✓	✓	✓	13 (93)	
Razavian et al [24], 2016	✓	✓	✓	✓	✓	✓	✓	N/A	✓	✓	✓	✓	✓	✓	13 (93)	
Richardson and Lidbury [25], 2017	✓	✓	✓	✓	✓	✓	✓	✓	✓	NR^d^	✓	✓	✓	✓	13 (93)	
Birks et al [26], 2017	✓	✓	✓	✓	✓	✓	✓	✓	CD	N/A	✓	✓	✓	✓	12 (86)	
Hernandez et al [27], 2017	✓	✓	✓	✓	✓	✓	CD	CD	✓	✓	✓	✓	✓	✓	12 (86)	
Roy et al [7], 2018	✓	✓	✓	✓	✓	✓	✓	CD	✓	✓	✓	✓	✓	✓	13 (93)	
Rawson et al [28], 2019	✓	✓	✓	✓	✓	✓	✓	✓	✓	N/A	✓	✓	✓	✓	13 (93)	
Aikens et al [29], 2019	✓	✓	✓	✓	✓	✓	✓	✓	✓	✓	✓	✓	✓	✓	14 (100)	
Hu et al [30], 2019	✓	✓	✓	✓	✓	✓	CD	N/A	✓	CD	✓	✓	✓	✓	11 (79)	
Bernardini et al [31], 2019	✓	✓	✓	✓	✓	✓	✓	✓	✓	✓	✓	✓	✓	✓	14 (100)	
Xu et al [32], 2019	✓	✓	✓	✓	✓	✓	✓	CD	✓	✓	✓	✓	✓	✓	13 (93)	
Lai et al [33], 2019	✓	✓	✓	✓	✓	✓	✓	✓	✓	N/A	✓	✓	✓	✓	13 (93)	
Tamune et al [34], 2020	✓	✓	✓	✓	✓	✓	CD	✓	✓	N/A	✓	✓	✓	✓	12 (86)	
Chicco and Jurman [35], 2020	✓	✓	✓	✓	✓	✓	✓	✓	✓	N/A	✓	✓	✓	✓	13 (93)	
Yu et al [36], 2020	✓	✓	✓	✓	✓	✓	✓	CD	NR	✓	✓	✓	✓	✓	12 (86)	
Banerjee et al [37], 2020	✓	✓	✓	✓	✓	✓	✓	N/A	✓	N/A	✓	✓	✓	✓	12 (86)	
Joshi et al [38], 2020	✓	✓	✓	✓	✓	✓	✓	N/A	CD	N/A	✓	✓	✓	✓	11 (79)	
Brinati et al [39], 2020	✓	✓	✓	✓	✓	✓	✓	N/A	✓	N/A	✓	✓	✓	✓	12 (86)	
Metsker et al [40], 2020	✓	✓	✓	✓	✓	✓	✓	✓	✓	N/A	✓	✓	✓	✓	13 (93)	
AlJame et al [41], 2020	✓	✓	✓	✓	✓	✓	✓	✓	✓	N/A	✓	✓	✓	✓	13 (93)	
Yadaw et al [42], 2020	✓	✓	✓	✓	✓	✓	✓	N/A	CD	N/A	✓	✓	✓	✓	11 (79)	
Cabitza et al [43], 2020	✓	✓	✓	✓	✓	✓	✓	N/A	✓	N/A	✓	✓	✓	✓	12 (86)	
Schneider et al [44], 2020	✓	✓	✓	✓	✓	✓	CD	✓	CD	N/A	✓	✓	✓	✓	11 (79)	
Yang et al [45], 2020	✓	✓	✓	✓	✓	✓	✓	✓	✓	N/A	✓	✓	✓	✓	13 (93)	
Plante et al [46], 2020	✓	✓	✓	✓	✓	✓	✓	CD	✓	N/A	✓✓	✓	✓	✓	12 (86)	
Mooney et al [47], 2020	✓	✓	✓	✓	✓	✓	✓	CD	✓	✓	✓	✓	✓	✓	13 (93)	
Yu et al [48], 2020	✓	✓	✓	✓	✓	✓	✓	✓	✓	N/A	✓	✓	✓	✓	13 (93)	
Kaftan et al [49], 2021	✓	✓	✓	✓	✓	✓	✓	✓	✓	N/A	✓	✓	✓	✓	13 (93)	
Park et al [50], 2021	✓	✓	✓	✓	✓	✓	CD	✓	CD	N/A	✓	✓	✓	✓	11 (79)	
Souza et al [51], 2021	✓	✓	✓	✓	✓	✓	✓	✓	✓	N/A	✓	✓	✓	✓	13 (93)	
Kukar et al [52], 2021	✓	✓	✓	✓	✓	✓	✓	✓	✓	N/A	✓	✓	✓	✓	13 (93)	
Gladding et al [53], 2021	✓	✓	✓	✓	✓	✓	✓	N/A	CD	N/A	✓	✓	✓	✓	11 (79)	
AlJame et al [41], 2021	✓	✓	✓	✓	✓	✓	✓	N/A	✓	N/A	✓	✓	✓	✓	12 (86)	
Rahman et al [54], 2021	✓	✓	✓	✓	✓	✓	✓	N/A	✓	N/A	✓	✓	✓	✓	12 (86)	
Myari et al [55], 2021	✓	✓	✓	✓	✓	✓	CD	✓	✓	✓	✓	✓	✓	✓	13 (93)	
Campagner et al [56], 2021	✓	✓	✓	✓	✓	✓	✓	✓	✓	N/A	✓	✓	✓	✓	13 (93)	
Babaei Rikan et al [57], 2022	✓	✓	✓	✓	✓	✓	✓	N/A	✓	N/A	✓	✓	✓	✓	12 (86)	

^a^Quality rating: ≥67%=good, 33% to 66%=fair, and ≤33%=poor.

^b^N/A: not applicable.

^c^CD: cannot be determined.

^d^NR: not reported.

**Table 2:** Description of the studies included in this review (N=40).

Author, year	Title	Description	Data set	Features	Methods	Main results
Richardson and Lidbury [21], 2013	Infection status outcome, machine learning method and virus type interact to affect the optimised prediction of hepatitis virus immunoassay results from routine pathology laboratory assays in unbalanced data	This study investigated the effect of data preprocessing, the use of ensembles constructed by bagging, and a simple majority vote to combine classification predictions from routine pathology laboratory data, particularly to overcome a significant imbalance of negative HBV^a^ and HCV^b^ cases HBV or HCV immunoassay positive cases.	Used a data set of 18,625 records from 1997 to 2007 made available by ACT Pathology at The Canberra Hospital, ACT^c^, Australia	Age, gender, and CBC^d^ (FBC^e^) parameters	Implemented the analysis using the RPART^f^ algorithm in R (DT^g^)	It was easier to predict positive immunoassay cases than negative cases of HBV or HCV.
Waljee et al [22], 2013	Comparison of imputation methods for missing laboratory data in medicine	Compare the accuracy of 4 imputation methods for missing entirely at random laboratory data and compare the effect of the imputed values on the accuracy of 2 clinical predictive models	The cirrhosis cohort had 446 patients, and the inflammatory bowel disease cohort had 395 patients from a tertiary-level care institution in Ann Arbor, Michigan.	CBC (FBC) parameters	MissForest, mean imputation, nearest neighbor imputation, and MICE^h^ to impute the simulated missing data	MissForest had the lowest imputation error for both continuous and categorical variables at each frequency of missingness, and it had the smallest prediction difference when models used imputed laboratory values.
Kinar et al [23], 2016	Development and validation of a predictive model for detection of colorectal cancer in primary care by analysis of complete blood counts: a binational retrospective study	Develop and validate a model to identify individuals at increased risk of CRC^i^	Used a data set of 2 million patients from the Maccabi Healthcare Services in Israel and the United Kingdom THIN^j^	Age, gender, and CBC (FBC) parameters	Gradient boosting model and RF^k^ classifier	Mean ROC AUC^l^ for detecting CRC was 0.82 (SD 0.01) for the Israeli validation set
Luo et al [5], 2016	Using Machine Learning to Predict Laboratory Test Results	Used ML^m^ to predict ferritin values from laboratory test results	Used a data set of 5128 inpatients in a tertiary care hospital in Boston, Massachusetts, collected over 3 months in 2013	Age, gender, and 41 laboratory tests	It used LR^n^, Bayesian LR, RFR^o^, and lasso regression (lasso).	The model could predict ferritin results with high accuracy (AUC^p^ as high as 0.97, held-out test data).
Razavian et al [24], 2016	Multi-task Prediction of Disease Onsets from Longitudinal Laboratory Tests	Using longitudinal measurements of laboratory tests, the study evaluated learning to predict disease onsets.	Used a data set from laboratory measurement and diagnosis information of 298,000 individuals from a larger cohort of 4.1 million insurance subscribers between 2005 and 2013	18 laboratory tests	The study trained an LSTM^q^ RNN^r^ and 2 novel CNNs^s^ for multitask prediction of disease onset.	These representation-based approaches significantly outperformed an LR with several hand engineered, clinically relevant features.
Richardson and Lidbury [25], 2017	Enhancement of hepatitis virus immunoassay outcome predictions in imbalanced routine pathology data by data balancing and feature selection before the application of support vector machines	The impact of 3 balancing methods and 1 feature selection method was explored to assess the ability of SVMs^t^ to classify imbalanced diagnostic pathology data associated with the laboratory diagnosis of HBV and HCV infections.	The data set used in this study originally comprised 18,625 individual cases of hepatitis virus testing over a decade, from 1997 to 2007.	Age, gender, and 26 laboratory tests	RFs	Generating data sets using the SMOTE^u^ resulted in significantly more accurate prediction than single downsizing or MDS^v^ of the data set.
Birks et al [26], 2017	Evaluation of a prediction model for colorectal cancer: retrospective analysis of 2.5 million patient records	Evaluate an existing risk algorithm derived in Israel that identifies individuals according to CRC risk using FBC data through CPRD^w^ data from the United Kingdom	2,550,119 patients who were ≥40 years old from CPRD	Age, gender, and CBC test	Application of the algorithm in case-control analysis of patients undergoing FBC testing during 2012 to estimate predictive values	The algorithm offered an additional means of identifying risk of CRC and could support other approaches to early detection, including screening and active case finding.
Hernandez et al [27], 2017	Supervised learning for infection risk inference using pathology data	Evaluated the performance of different binary classifiers to detect any type of infection from a reduced set of commonly requested clinical measurements	Pathology and microbiology data of patients from all hospital wards at ICHNT^x^ were extracted.	Alanine aminotransferase, alkaline phosphatase, bilirubin, creatinine, C-reactive proteins, and WBC^y^	Supervised ML algorithms for binary classification (Gaussian NB^z^, DT classifier, RF classifier, and SVM)	ROC AUC (0.80-0.83), sensitivity (0.64-0.75), and specificity (0.92-0.97)
Roy et al [7], 2018	Predicting Low Information Laboratory Diagnostic Tests	The study described the prevalence of common laboratory tests in a hospital environment and the rate of “normal” results to quantify pretest probabilities under different conditions.	Electronic medical records (Epic) of 71,000 patients admitted to Stanford Tertiary Academic Hospital between the years 2008 and 2014	Common laboratory tests (eg, thyroid stimulating hormone, sepsis protocol lactate, ferritin, and NT-PROBNP^aa^)	Provided a data-driven, systematic method to identify cases where the incremental value of testing is worth reconsidering	The study found that low-yield laboratory tests were common (eg, approximately 90% of blood cultures were normal).
Rawson et al [28], 2019	Supervised machine learning for the prediction of infection on admission to hospital: A prospective observational cohort study	An SML^ab^ algorithm was developed to classify cases into infection versus no infection using microbiology records and 6 available blood parameters.	This study took place at ICHNT, comprising 3 university teaching hospitals. The study took place between October 2017 and March 2018 with 160,203 individuals.	C-reactive protein, WCC^ac^, bilirubin, creatinine, ALT^ad^, and alkaline phosphatase	A (SVM) binary classifier algorithm was developed and incorporated into the EPIC IMPOC^ae^ CDSS^af^ for investigation within this study following validation and pilot assessment.	The infection group had a likelihood of 0.80 (SD 0.09), and the noninfection group had a likelihood of 0.50 (0.29, 95% CI 0.20-0.40; *P*<.01). ROC AUC was 0.84 (95% CI 0.76-0.91).
Aikens et al [29]	A machine learning approach to predicting the stability of inpatient lab test results	Development of a predictive model that can identify low-information laboratory tests before they are ordered	Analyzed 6 years (2008-2014) of inpatient data from Stanford University Hospital, a tertiary academic hospital	Troponin, thyroid stimulating hormone, platelet count, phosphate in serum or plasma, partial thromboplastin time, NT-PROBNP, magnesium, lipase, lactase, heparin activity, ferritin, creatinine kinase, and C-reactive protein	Six different ML models for classification: a DT, a boosted tree classifier (AdaBoost), an RF, a Gaussian NB classifier, a lasso-regularized LR, and a linear regression followed by rounding to 0 or 1	A large proportion of repeat tests were within an SD of 10% or 0.1 of the previous measurement, indicating that a large volume of repetitive testing may be contributing little new information.
Hu et al [30], 2019	Using Biochemical Indexes to Prognose Paraquat-Poisoned Patients: An Extreme Learning Machine-Based Approach	Explore useful indexes from biochemical tests and identify their predictive value in prognosis of patients poisoned with PQ^ag^	The biochemical indexes of 101 patients poisoned with PQ who were hospitalized in the emergency room of First Affiliated Hospital of Wenzhou Medical University from 2013 to 2017	Total bilirubin, direct bilirubin, indirect bilirubin, total protein, albumin, albumin-globulin ratio, alanine aminotransferase, aspartate aminotransferase, the ratio of AST^ah^ to ALT, blood glucose, urea nitrogen, and creatinine	An effective ELM^ai^ model was developed for classification tasks.	A new method for prognosis of PQ poisoning with accuracy of 79.6%
Bernardini et al [31], 2019	TyG-er: An ensemble Regression Forest approach for identification of clinical factors related to insulin resistance condition using Electronic Health Records	The study aimed to discover nontrivial clinical factors in EHR^aj^ data to determine where the insulin resistance condition is encoded.	A total of 2276 records from 968 patients not affected by T2D^ak^; the longitudinal patient observational period was from 2010 to 2018 (FIMMG_obs data set)	Gender, age, blood pressure, height, weight, and 73 laboratory exams	Highly interpretable ML approach (ie, ensemble regression forest combined with data imputation strategies), named TyG-er	High agreement (from 0.664 to 0.911 of the Lin correlation coefficient) of the TyG-er and predictive power of the TyG-er approach (up to a mean absolute error of 5.68% and correlation coefficient=0.666; *P*<.05)
Xu et al [32], 2019	Prevalence and Predictability of Low-Yield Inpatient Laboratory Diagnostic Tests	Identify inpatient diagnostic laboratory testing with predictable results that are unlikely to yield new information	A total of 116,637 inpatients treated at Stanford University Hospital from January 2008 to December 2017; 60,929 inpatients treated at the University of Michigan from January 2015 to December 2018; and 13,940 inpatients treated at the University of California, San Francisco from January 2018 to December 2018 were assessed.	The core features included patient demographics, change of the most recent test, number of recent tests, history of Charlson Comorbidity Index categories, which specialty team was treating the patient, time since admission, statistical data, and laboratory test results.	Regularized LR, regression and round, NB, NN^al^ multilayer perceptrons, DT, RF, AdaBoost, and XGB^am^	The findings suggest that low-yield diagnostic testing is common and can be systematically identified through data-driven methods and patient context–aware predictions.
Lai et al [33], 2019	Predictive models for diabetes mellitus using machine learning techniques	The objective of this study was to build an effective predictive model with high sensitivity and selectivity to better identify Canadian patients at risk of having diabetes mellitus based on patient demographic data and the laboratory test results during their visits to medical facilities.	13,309 Canadian patients aged between 18 and 90 years	Age, sex, fasting blood glucose, BMI, high-density lipoprotein, triglycerides, blood pressure, and low-density lipoprotein	Predictive models using LR and GBM^an^ techniques	The ROC AUC for the proposed GBM model was 84.7% with a sensitivity of 71.6%, and the ROC AUC for the proposed LR model was 84% with a sensitivity of 73.4%.
Tamune et al [34], 2020	Efficient Prediction of Vitamin B Deficiencies via Machine-Learning Using Routine Blood Test Results in Patients with Intense Psychiatric Episode	Predict vitamin B deficiency using ML models from patient characteristics and routine blood test results that can be obtained within 1 hour	Reviewed 497 patients admitted to the Department of Neuropsychiatry at Tokyo Metropolitan Tama Medical Center between September 2015 and August 2017	Age, sex, and 29 routine blood tests	ML models (KNN^ao^, LR, SVM, and RF)	The study demonstrated that ML can efficiently predict some vitamin deficiencies in patients with active psychiatric symptoms.
Chicco and Jurman [35], 2020	Machine learning can predict survival of patients with heart failure from serum creatinine and ejection fraction alone	ML in particular can predict patients’ survival from their data and individuate the most important features among those included in their medical records.	Medical records of 299 patients with heart failure collected at the Faisalabad Institute of Cardiology and the Allied Hospital in Faisalabad (Punjab, Pakistan) from April 2015 to December 2015	Age, anemia, high blood pressure, creatinine phosphokinase, diabetes, ejection fraction, sex, platelets, serum creatinine, serum sodium, smoking, and follow-up period	Apply several ML classifiers to both predict the patient’s survival and rank the features corresponding to the most important risk factors	The results of these 2-feature models show not only that serum creatinine and ejection fraction are sufficient to predict survival of patients with heart failure from medical records but also that using these 2 features alone can lead to more accurate predictions than using the original data set features in their entirety.
Yu et al [36], 2020	Predict or draw blood: An integrated method to reduce lab tests	Propose a novel DL^ap^ method to jointly predict future laboratory test events to be omitted	The data set (MIMIC III) contained 598,444 laboratory test results and 5,598,079 vital sign records from a total of 41,113 adult patients (aged ≥16 years) admitted to critical care units between 2001 and 2012.	Sodium, potassium, chloride and serum bicarbonate, total calcium, magnesium, phosphate, BUN^aq^, creatinine, hemoglobin, platelet count, and WBC.	The study ran a novel DL method combining 4 features: lab (laboratory test data), D (demographic data), V (vital data, which were mean and SD in the vicinity of the corresponding laboratory tests), and C (encoding to indicate missing values).	Was able to omit 15% of laboratory tests with <5% prediction accuracy loss
Banerjee et al [37], 2020	Use of Machine Learning and Artificial Intelligence to predict SARS-CoV-2 infection from Full Blood Counts in a population	The aim of the study was to use ML, an ANN^ar^, and a simple statistical test to identify patients who were SARS-CoV-2–positive from FBCs without knowledge of symptoms or history of the individuals.	The data set included in the analysis and training contained anonymized FBC results from 5664 patients seen at the Hospital Israelita Albert Einstein (São Paulo, Brazil) from March 2020 to April 2020 and who had samples collected to perform the SARS-CoV-2 RT-PCR^as^ test during a visit to the hospital.	Age and CBC (FBC) parameters	RF and lasso-based regularized generalized linear models and ANN	The study found that, with FBCs, RF, shallow learning, and a flexible ANN model predict patients with SARS-CoV-2 with high accuracy between populations on regular wards (AUC=94%-95%) and those not admitted to the hospital or in the community (AUC=80%-86%).
Joshi et al [38], 2020	A predictive tool for identification of SARS-CoV-2 PCR-negative emergency department patients using routine test results	Predict SARS-CoV-2 PCR^at^ positivity based on CBC components and patient sex	357 CBC data from January 2020 to March 2020 ordered within 24 hours of a SARS-CoV-2 PCR test (based off the WHO^au^ assay)	Absolute neutrophil count, absolute lymphocyte count, and hematocrit	The study trained an L2^av^-regularized LR model.	Prediction of SARS-CoV-2 PCR positivity demonstrated a C-statistic of 78% and an optimized sensitivity of 93%.
Brinati et al [39], 2020	Detection of COVID-19 Infection from Routine Blood Exams with Machine Learning: A Feasibility Study	Develop a predictive model based on ML techniques to predict positivity or negativity for COVID-19	Data set available from the IRCCS^aw^ Ospedale San Raffaele 2 with 279 cases randomly extracted from the end of February 2020 to mid-March 2020	Gender, age, leukocytes, platelets, C-reactive protein, transaminases, gamma-glutamyltransferase, lactate dehydrogenase, neutrophils, lymphocytes, monocytes, eosinophils, and basophils	DT, ETs^ax^, KNN, LR, NB, RF, and SVMs	Their accuracy ranged from 82% to 86%, and sensitivity ranged from 92% to 95%.
Metsker et al [40], 2020	Identification of risk factors for patients with diabetes: diabetic polyneuropathy case study	Implementation of ML methods for identifying the risk of diabetes polyneuropathy based on structured electronic medical records collected from databases of medical information systems	Laboratory records from 5425 patients between 2010 and 2017	16 laboratory tests plus a CBC	ANN, SVM, DT, linear regression, and LR classifier	79.82% precision, 81.52% recall, 80.64% *F*_1_-score, 82.61% accuracy, and 89.88% AUC using the NN classifier
AlJame et al [41], 2020	Ensemble learning model for diagnosing COVID-19 from routine blood tests	The study proposed ERLX, which is an ensemble learning model for COVID-19 diagnosis from routine blood tests.	The study used 5644 data samples with 559 confirmed COVID-19 cases from a publicly available data set from Albert Einstein Hospital in Brazil.	24 laboratory tests, including INR^ay^, albumin, D-dimer, and prothrombin time	The proposed model used 3 classifiers—extra trees, RF, and LR—combining their predictions with an XGB.	The ensemble model achieved outstanding performance, with an overall accuracy of 99.88%, AUC of 99.38%, sensitivity of 98.72%, and specificity of 99.99%.
Yadaw et al [42], 2020	Clinical Predictive Models for COVID-19: Systematic Study	The aim of this study was to develop, study, and evaluate clinical predictive models that estimate, using ML and based on routinely collected clinical data, which patients are likely to receive a positive SARS-CoV-2 test or require hospitalization or intensive care.	The study used anonymized data from a cohort of 5644 patients seen at the Hospital Israelita Albert Einstein in São Paulo, Brazil, in the early months of 2020.	The study used 106 routine clinical, laboratory, and demographic measurements.	LR, NN, RF, SVM, and gradient boosting (XGB)	Predicted positive tests for SARS-CoV-2 a priori at a sensitivity of 75% and a specificity of 49%, patients who were SARS-CoV-2–positive who required hospitalization with 0.92 AUC, and patients who were SARS-CoV-2–positive who required critical care with 0.98 AUC
Cabitza et al [43], 2020	Development, evaluation, and validation of machine learning models for COVID-19 detection based on routine blood tests	Routine blood tests can be exploited using the authors’ method to diagnose COVID-19.	1925 patients on admission to the ED^az^ at the San Raffaele Hospital (OSR^ba^) from February 2020 to May 2020	72 features: CBC, biochemical, coagulation, hemogas analysis and CO-oximetry values, age, sex, and specific symptoms at triage	RF, NB, LR, SVM, and KNN	For the complete OSR data set, the AUC for the algorithms ranged from 0.83 to 0.90; for the COVID-19–specific data set, it ranged from 0.83 to 0.87.
Schneider et al [44], 2020	Validation of an Algorithm to Identify Patients at Risk for Colorectal Cancer Based on Laboratory Test and Demographic Data in Diverse, Community-Based Population	Validate a predictive score generated by an ML algorithm with common laboratory test data to identify patients at high risk of CRC in a large, community-based, ethnically diverse cohort	The eligible study cohort population included 2,855,994 KPNC^bb^ Health Plan members between 1996 and 2015.	Gender, year of birth, and at least one CBC test, including cell parameters	Validate the ability of an algorithm that uses laboratory and demographic information to identify patients at increased risk of CRC	The algorithm identified 3% of the population who required an investigation and 35% of patients who received a diagnosis of CRC within the following 6 months.
Yang et al [45], 2020	Routine Laboratory Blood Tests Predict SARS-CoV-2 Infection Using Machine Learning	Develop an ML model integrating age, gender, race, and routine laboratory blood tests, which are readily available with a short TAT^bc^	5893 patients evaluated at the NYPH^bd^ and WCM^be^ from March 2020 to April 2020	26 laboratory tests, including C-reactive protein, ferritin, lactic acid dehydrogenase, and magnesium	Used a GBDT^bf^ model	The model achieved an AUC of 0.854. The model, too, predicted initial SARS-CoV-2 RT-PCR positivity in 66% of individuals whose RT-PCR result changed from negative to positive within 2 days.
Plante et al [46], 2020	Development and External Validation of a Machine Learning Tool to Rule Out COVID-19 Among Adults in the Emergency Department Using Routine Blood Tests: A Large, Multicenter, Real-World Study	Develop an ML model to rule out COVID-19 using only routine blood tests among adults in EDs	Model training used 2183 PCR-confirmed cases from 43 hospitals during the pandemic; negative controls were 10,000 prepandemic patients from the same hospitals. External validation used 23 hospitals with 1020 PCR-confirmed cases and 171,734 prepandemic negative controls.	14 laboratory tests, including sodium, bicarbonate, BUN, and chloride	XGB ML model	The model found high discrimination across age, race, sex, and disease severity subgroups and had high diagnostic yield at low score cutoffs in a screening population with a disease prevalence of <10%. Such a model could rapidly identify those at low risk of COVID-19 in a “rule out” method and might reduce the need for PCR testing in such patients.
Mooney et al [47], 2020	Predicting bacteraemia in maternity patients using full blood count parameters: A supervised machine learning algorithm approach	Use ML tools to identify if bacteremia in pregnant or postpartum women could be predicted using FBC parameters other than the WCC	129 women from the Rotunda Hospital in 2019, a stand-alone tertiary-level maternity hospital in Ireland	WCC, absolute neutrophils, lymphocytes, monocytes, eosinophils, basophils, NLR^bg^, platelets, MPV^bh^, MPV to platelet ratio, and monocyte to lymphocyte ratio	LDA^bi^, KNN, SVM with a linear kernel, and RF along with CART^bj^	Sensitivity of 27.9% (95% CI 20.3-36.4), specificity of 94.1% (95% CI 93.3-94.8), PPV^bk^ of 13.9% (95% CI 10.6-17.9), and NPV^bl^ of 97.4% (95% CI 97.2-97.7)
Yu et al [48], 2020	A deep learning solution to recommend laboratory reduction strategies in ICU	Build an ML model that predicts laboratory test results and provides a promising laboratory test reduction strategy using spatial-temporal correlations	The Medical Information Mart for Intensive Care III data set with 53,423 distinct hospital admissions of adult patients to intensive care units at Beth Israel Deaconess Medical Center	Sodium, potassium, chloride, serum bicarbonate, total calcium, magnesium, phosphate, BUN, creatinine, hemoglobin, platelet count, WBC, age, gender, and race	Built a DL model with 5 variants for each of the combinations of input features	The model predicted normality or abnormality of laboratory tests with a 98.27% accuracy (AUC=0.9885; sensitivity 97.84%; specificity 98.8%; PPV=99.01%; NPV=97.39%) on 20.26% reduced laboratory tests and recommended 98.1% of transitions to be checked.
Kaftan et al [49], 2021	Predictive Value of C-reactive Protein, Lactate Dehydrogenase, Ferritin and D-dimer Levels in Diagnosing COVID-19 Patients: a Retrospective Study	The study aimed to evaluate the diagnostic accuracy of CRP^bm^, ferritin, LDH^bn^, and D-dimer in predicting positive cases of COVID-19 in Iraq.	The sample size was based on a minimum sensitivity and specificity of 95%; the study randomly selected medical records of 938 patients suspected to have COVID-19 between May 2020 and December 2020.	Age, gender, C-reactive protein, ferritin, LDH, and D-dimer.	A retrospective observational cohort study based on STARD^bo^ guidelines to determine the diagnostic accuracy of COVID-19	A combination of routine laboratory biomarkers (CRP, LDH, and ferritin ±D-dimer) can be used to predict the diagnosis of COVID-19 with an accepted sensitivity and specificity before proceeding to definitive diagnosis through RT-PCR.
Park et al [50], 2021	Development of machine learning model for diagnostic disease prediction based on laboratory tests	Build a new optimized ensemble model by blending a DNN^bp^ model with 2 ML models for disease prediction using laboratory test results	The study analyzed data sets provided by the Department of Internal Medicine from 5145 patients visiting the emergency room and those admitted to Catholic University of Korea St. Vincent’s Hospital in Suwon, Korea, between 2010 and 2019.	The study confirmed a total of 88 attributes, including sex and age.	The study developed a new ensemble model by combining their DL (DNN) model with their 2 ML models (SVM and RF) to improve AI^bq^ performance.	The optimized ensemble model achieved an *F*_1_-score of 81% and a prediction accuracy of 92% for the 5 most common diseases.
Souza et al [51], 2021	Simple hemogram to support the decision-making of COVID-19 diagnosis using clusters analysis with self-organising maps neural network	Identify potential variables in routine blood tests that can support clinician decision-making during COVID-19 diagnosis at hospital admission	5644 patients allocated to the Albert Einstein Hospital in São Paulo, Brazil, in the Kaggle platform on March 2020	14 variables present in the blood test	Nonsupervised clustering analysis with NN SOM^br^ as a strategy of decision-making	It was possible to detect a group of units of the map with a discrimination power of approximately 83% to patients who were SARS-CoV-2–positive.
Kukar et al [52], 2021	COVID-19 diagnosis by routine blood tests using machine learning	The aim of this study was to determine the diagnostic accuracy of an ML model built specifically for the diagnosis of COVID-19 using the results of routine blood tests.	52,306 patients admitted to the Department of Infectious Diseases, UMCL^bs^, Slovenia, in March 2020 and April 2020	Age, gender, and 35 laboratory tests	SBA^bt^ algorithm: a CRISP-DM^bu^–based ML pipeline consisting of 5 processing stages and using an XGB model	The model exhibited a high sensitivity of 81.9%, a specificity of 97.9%, and an AUC of 0.97.
Gladding et al [53], 2021	A machine learning PROGRAM to identify COVID-19 and other diseases from haematology data	The study proposed a method for screening FBC metadata for evidence of communicable and noncommunicable diseases using ML.	A total of 156,570 hematology raw data were collected between July 2019 and June 2020 from Waitakere Hospital and North Shore Hospital.	A maximum of 247 FBC features from CSV^bv^ data were used; 134 were categorical, and 101 were numeric.	MDCalc software was used to analyze and apply ML models using DTs and ensembles, LR, and DNNs.	Urinary tract infection: ROC AUC=0.68, sensitivity=52%, and specificity=79%; COVID-19: ROC AUC=0.8, sensitivity=82%, and specificity=75%; heart failure: ROC AUC=0.78, sensitivity=72%, and specificity=72%
AlJame et al [41], 2021	Deep forest model for diagnosing COVID-19 from routine blood tests	Develop an ML prediction model to accurately diagnose COVID-19 from clinical or routine laboratory test data	5644 patient records that were collected from March 2020 to April 2020 (Albert Einstein Israelita Hospital, located in São Paulo, Brazil) and 279 patients who were admitted to San Raffaele Hospital, Milan, Italy, from the end of February 2020 to mid-March 2020	Age, gender, and 13 laboratory tests	DF^bw^ model constructed from 3 different classifiers: extra trees, XGB, and LightGBM	Experimental results show that the proposed DF model has an accuracy of 99.5%, sensitivity of 95.28%, and specificity of 99.96%.
Rahman et al [54], 2021	Mortality Prediction Utilising Blood Biomarkers to Predict the Severity of COVID-19 Using Machine Learning Technique	Development of a prediction model of high mortality risk for patients both with and without COVID-19	654 patients with and without COVID-19 were admitted to the ED in Boston (March 2020 to April 2020) and Tongji Hospital in China (January 2020 to February 2020).	Age, lymphocyte count, D-dimer, CRP, and creatinine	RF, SVM, KNN, XGB, extra trees, and LR	For the development cohort and the internal and external validation cohorts using LR, the AUCs were 0.987, 0.999, and 0.992, respectively.
Myari et al [55], 2021	Diagnostic value of white blood cell parameters for COVID‐19: Is there a role for HFLC and IG?	Investigate the ability of WBC and its subsets, HFLC^bx^, IG^by^, and C-reactive protein to aid diagnosis of COVID-19 during the triage process and as indicators of disease progression to serious and critical condition	A retrospective case-control study conducted with data collected from patients admitted to the ED of University General Hospital of Ioannina (Ioannina, Epirus, Greece) from March 2020 to March 2021	Age, gender, and 13 laboratory tests	Enter binary LR analysis was conducted to determine the influence of the parameters on the outcome.	The combined WBC-HFLC marker was the best diagnostic marker for both mild and serious disease. CRP and lymphocyte count were early indicators of progression to serious disease, whereas WBC, NEUT^bz^, IG, and the NLR were the best indicators of critical disease.
Campagner et al [56], 2021	External validation of Machine Learning models for COVID-19 detection based on Complete Blood Count	Evaluate whether ML models for COVID-19 diagnosis based on CBC data could be robust to cross-site transportability and, thus, could be reliably deployed as medical decision support tools	Data from 1736 patients collected at the EDs of the IRCCS Hospital San Raffaele and the IRCCS Istituto Ortopedico Galeazzi of Milan (Italy)	Age, gender, and 23 routine laboratory tests	RF, LR, KNN, SVM, NB, and ensemble	The study reported an average AUC of 95%. The best-performing model (SVM) reported an average AUC of 97.5%.
Babaei Rikan et al [57], 2022	COVID-19 diagnosis from routine blood tests using artificial intelligence techniques	The study presented the development and comparison of various models for diagnosing positive cases of COVID-19 using 3 data sets of routine laboratory blood tests.	A total of 3 open-access study data sets from 2498 patients containing routine blood test data from COVID-19 and non–COVID-19 cases were used.	Routine laboratory tests according to each of the 3 data sets	Seven ML methods —LR, KNN, DT, SVM, NB, ET, RF. In addition to XGB —along with 4 DL methods: DNN, CNN, RNN, and LSTM	On average, accuracy, specificity, and AUC were 92.11%, 84.56%, and 92.2% for the first data set; 93.16%, 93.02%, and 93.2% for the second data set; and 92.5%, 85%, and 92.2% for the third data set, respectively.

^a^HBV: hepatitis B virus.

^b^HCV: hepatitis C virus.

^c^ACT: Australian Capital Territory.

^d^CBC: complete blood count.

^e^FBC: full blood count.

^f^RPART: Recursive Partitioning.

^g^DT: decision tree.

^h^MICE: Multivariate Imputation by Chained Equations.

^i^CRC: colorectal cancer.

^j^THIN: The Health Improvement Network.

^k^RF: random forest.

^l^ROC AUC: area under the receiver operating characteristic curve.

^m^ML: machine learning.

^n^LR: logistic regression.

^o^RFR: RF regression.

^p^AUC: area under the curve.

^q^LSTM: long short-term memory.

^r^RNN: recurrent neural network.

^s^CNN: convolutional neural network.

^t^SVM: support vector machine.

^u^SMOTE: Synthetic Minority Over-sampling Technique.

^v^MDS: multiple downsizing.

^w^CPRD: Clinical Practice Research Datalink.

^x^ICHNT: Imperial College Healthcare National Health Service Trust.

^y^WBC: white blood count.

^z^NB: naïve Bayes.

^aa^NT-PROBNP: N-terminal pro–brain natriuretic peptide.

^ab^SML: supervised machine learning.

^ac^WCC: white cell count.

^ad^ALT: alanine aminotransferase.

^ae^EPIC IMPOC: Enhanced, Personalized, and Integrated Care for Infection Management at the Point-of-Care.

^af^CDSS: clinical decision support system.

^ag^PQ: Paraquat.

^ah^AST: aspartate transaminase.

^ai^ELM: extreme learning machine.

^aj^EHR: electronic health record.

^ak^T2D: type 2 diabetes.

^al^NN: neural network.

^am^XGB: extreme gradient boosting.

^an^GBM: gradient boosting machine.

^ao^KNN: k-nearest neighbor.

^ap^DL: deep learning.

^aq^BUN: blood urea nitrogen.

^ar^ANN: artificial NN.

^as^RT-PCR: reverse transcription polymerase chain reaction.

^at^PCR: polymerase chain reaction.

^au^WHO: World Health Organization.

^av^L2: L2-penalization.

^aw^IRCCS: Scientific Institute for Research, Hospitalization and Healthcare.

^ax^ET: extremely randomized trees.

^ay^INR: international normalized ratio.

^az^ED: emergency department.

^ba^OSR: San Raphael Hospital.

^bb^KPNC: Kaiser Permanente Northern California.

^bc^TAT: turnaround time.

^bd^NYPH: New York Presbyterian Hospital.

^be^WCM: Weill Cornell Medicine.

^bf^GBDT: gradient boosting DT.

^bg^NLR: neutrophil to lymphocyte ratio.

^bh^MPV: mean platelet volume.

^bi^LDA: linear discriminant analysis.

^bj^CART: classification and regression trees.

^bk^PPV: positive predictive value.

^bl^NPV: negative predictive value.

^bm^CRP: C-reactive protein.

^bn^LDH: lactate dehydrogenase.

^bo^STARD: Standards for the Reporting of Diagnostic Accuracy Studies.

^bp^DNN: deep NN.

^bq^AI: artificial intelligence.

^br^SOM: self-organizing map.

^bs^UMCL: University Medical Centre Ljubljana.

^bt^SBA: Smart Blood Analytics.

^bu^CRISP-DM: cross-industry process for data mining.

^bv^CSV: comma-separated value.

^bw^DF: deep forest.

^bx^HFLC: high-fluorescence lymphocyte cell.

^by^IG: immature granulocyte count.

^bz^NEUT: neutrophil count.

## Discussion

### Principal Findings

This study aimed to identify studies that used laboratory tests to predict new results. Our interest in this line of study was motivated by the possibility that laboratory tests can be used more comprehensively to search for hidden information, discovering previously unknown pathologies. This methodology is highly advantageous for the diagnostic process of medical laboratories. In this sense, intelligent systems could automatically analyze the examinations performed on a patient and make predictions in the search for hidden pathologies. In positive cases, alarms would be generated, and complementary examinations would be suggested. In most cases, the collected sample could be used to carry out new tests.

The use of laboratory tests to predict results has been increasingly explored. In recent years, several studies have obtained good results using clinical data to search for diagnoses [58]. In addition to laboratory tests, the studies in this review used patient histories, imaging tests, and medical diagnoses. For example, Wu et al [59] and Hische et al [60], in addition to laboratory tests, also made use of other clinical data in the search for a diagnosis. Some studies, such as those by Ravaut et al [61] and Le et al [62], aimed to determine whether a patient was likely to develop the disease in the future, which is quite relevant as part of a process in predictive medicine. These studies obtained good results but used clinical or diagnostic data. This information is generated through the analysis by a physician, unlike most laboratory tests such as the complete blood count, which follows an automated analytical process without the intervention of human factors.

However, in this research, we only looked for studies that emphasized laboratory tests to predict new information. This methodology can innovate the diagnostic processes of medical laboratories and has attracted the interest of several researchers over time, especially in recent years owing to the COVID-19 pandemic. In total, we found 40 studies referring to the last decade that met the established criteria, with most studies published in 2020 (15/40, 38%) and 2021 (10/40, 25%).

All (40/40, 100%) the studies presented in this review used laboratory tests as input data in addition to some clinical data such as gender and age. Some (12/40, 30%) studies used >20 parameters, such as the study by Yadaw et al [42], who used >100 different parameters. Others (6/40, 15%) used very few parameters, as is the case of the work by Joshi et al [38], who used only 3 parameters (absolute neutrophil count, absolute lymphocyte count, and hematocrit). However, most (22/40, 55%) studies used approximately 10 parameters, with the complete blood count as the primary data source. Finally, 22% (9/40) of the studies used full blood count data only.

When analyzing the quality assessment tool (Table 1), all studies showed good results, with an average value of 88%. As most of the studies were characterized as retrospective cohort studies, the data used were generated before the research. Thus, questions 8 and 10 of the questionnaire [19], referring to the levels and amount of exposure, were answered mainly with *not applicable* or *cannot be determined*. This fact lowered the average slightly in the evaluation process of most (38/40, 95%) studies. However, 5% (2/40) of the studies [29,31] were evaluated with 100%. Another 45% (18/40) of the studies were evaluated with 93%, 32% (13/40) of the studies were evaluated with 86%, and 18% (7/40) of the studies were evaluated with 79%.

Table 2 presents a summary of the main characteristics of the studies. In addition to a brief description of the research, it is possible to know the methodology and the main results in a simplified way.

It is not possible to make a comparison between the methodology and results of the selected studies as they had different objectives. Our goal was to confirm the possibility of predicting specific examinations from other examinations and which ML methods and parameters were most used.

Regarding the models, most (39/40, 98%) studies used ML methods with supervised training, almost always aiming at the exam responsible for the diagnosis. Of the 40 studies selected, only 3 (8%) used regression methods, whereas the other 37 (92%) used classification methods. Among the most used models, we can mention logistic regression, random forest, support vector machine, and k-nearest neighbor, trained as binary classifiers. In the case of neural networks, they were almost always used with deep learning techniques (deep neural networks [DNNs]).

The random forest method was the most tested, with 50% (20/40) of the studies using it. The next most tested methods were logistic regression with 45% (18/40) of the studies and support vector machine with 35% (14/40) of the studies, followed by naïve Bayes, decision tree, and XGBoost with 25% (10/40) of the studies each. By contrast, artificial neural networks were tested in 18% (7/40) of the studies, in addition to DNN methods in another 15% (6/40) of the studies.

In general, the most efficient method was the DNN, such that, of the 6 studies that used this method, 5 (83%) had better results with it. Next, there was the XGBoost method, such that, of the 10 studies that used this method, 7 (70%) considered it better, followed by random forest, where, of the 20 studies that tested this method, 12 (60%) had better results with it. In a simplified way, we can say that the DNN method was 83% better than the others, followed by XGBoost (70% better) and random forest (60% better).

Although the DNN model presents better results, the random forest method is quite attractive, not only because it is simple and fast but also because it presents the path taken in the search for the result, which is quite relevant in research in the health care domain.

Research that initially caught our attention was conducted by Luo et al [5] to predict ferritin levels to detect patients with anemia. The research used 41 laboratory tests from 989 patients admitted to the tertiary care hospital in Boston, Massachusetts, for 3 months in 2013. The work had good results, with an area under the curve (AUC) of 97%. The most interesting thing is that, even in cases where the ferritin tests were false negatives, the system could detect anemia. This result shows that laboratory tests may have more information when analyzed holistically than when referring to the specific test performed.

Rawson et al [28] used laboratory tests to identify cases of bacterial infection among 160,203 hospitalized patients over 6 months. An interesting feature of this research is that only 6 tests were used as input parameters (C-reactive protein, white blood cell count, bilirubin, creatinine, alanine aminotransferase, and alkaline phosphatase), achieving good results, with an area under the receiver operating characteristic curve of 0.84. The use of a low number of examinations was an important factor in building the model. This situation makes it possible to use tests already performed on patients, making the screening process fast and straightforward without collecting more blood samples from a patient.

Of the selected studies, 8% (3/40) focused on the prediction of colorectal cancer. Colorectal cancer has a high incidence rate, accounting for many deaths worldwide. The early identification of this type of pathology can be very advantageous to governments and health systems, who can provide adequate treatment to prevent the worsening of the disease. Kinar et al [23] obtained good results in identifying patients with a propensity to develop colorectal cancer 1 year before the development of the disease. In this study, 20 parameters from the complete blood count of approximately 2 million patients were used. Similarly, Birks et al [26] used the complete blood count of 2.5 million patients, obtaining an AUC of 75% for more extended periods (3 years) and 85% for shorter periods (6 months). More recently, Schneider et al [44] also obtained a mean AUC of 78% in a study of approximately 2.8 million patients seen between 1996 and 2015.

Another 12% (5/40) of the studies [7,29,32,36,48] aimed to identify tests that would not change over time, remaining classified as normal without the need to be repeated. In general, all of them showed good results; however, we highlight the work by Xu et al [32], who obtained an AUC of >90% for 12 months of analysis.

A recent publication that also caught our attention was the work by Park et al [50]. The authors used deep learning models to predict 39 different diseases in their research, reaching an accuracy of >90% and an *F*_1_-score of 81% for the 5 most common diseases. They used 88 features from 5145 patients who visited the emergency room.

The use of laboratory tests and ML techniques has increased in recent years, mainly owing to the COVID-19 pandemic. Of the 40 studies in this review, 27 (68%) published between 2020 and 2022 were selected. Of these 27 studies, 19 (70%) studies were related to SARS-CoV-2, a total of 8 (30%) studies were published in 2020, a total of 9 (33%) studies were published in 2021, and 1 (4%) study was published in 2022. All of them used laboratory tests to predict some unknown information, and most (34/40, 85%) studies focused on the search for a diagnosis.

Analyzing aspects related to training and the potential for bias based on the data sets, a common feature among most studies was the fact that 92% (37/40) of them were treated as a classification problem using supervised models. In this process, a point to be considered is the fact that the target classes of the models are almost always defined by a medical diagnosis or a reference value. In class prediction, the results of values close to the classification margins may be affected, influencing the final result of the model.

Another aspect that draws attention is the fact that the data sets were highly unbalanced, with some (3/40, 8%) studies [21,23,26] where the target represented <1% of the data set, implying some care to avoid errors in the training and evaluation process. In this sense, most (34/40, 85%) of the analyzed studies used the area under the receiver operating characteristic curve as the main evaluation metric, with an average value of approximately 85%. Although this metric is quite common in health-related problems, some authors defend [63] the use of the area under the precision-recall curve as the most appropriate metric for strongly unbalanced bases.

Considering the aspects discussed, we question whether, in the search for a diagnosis, it would not be more appropriate to treat the prediction of new tests as a regression problem, leaving the responsibility of decision-making to health professionals.

### Limitations

One of the limitations of this study was how the articles were selected, analyzing only the data from the titles, keywords, and abstracts initially reviewed.

Another limitation was the nonuse of studies whose data source consisted of imaging examinations and clinical history and where the objective was not a prediction.

These criteria greatly reduced the number of selected studies. However, our objective was to analyze only studies that had a main focus on the use of laboratory tests. These requirements are fundamental in building models that can automatically analyze test results without affecting the processes of medical laboratories.

### Conclusions

In the search for scientific research that used laboratory tests and ML models to predict new information, 40 studies were found that fit the established criteria. Among these, all (40/40, 100%) sought to predict unknown information, with most (34/40, 85%) focused on the search for a diagnosis.

We have seen a large increase in the use of this methodology in recent years, mainly motivated by the COVID-19 pandemic. Of the 40 works selected from 2010 onward, 27 (68%) focused on SARS-CoV-2, published between 2020 and 2022.

All (40/40, 100%) studies used only laboratory tests, and the complete blood count was the most used. The use of routine examinations is encouraged, mainly as they are more frequently performed and have greater availability. Among the prediction methods, most (39/40, 98%) studies used ML models with supervised learning. These techniques have been spreading and obtaining good results over the years, and binary classification models are still the most used, with XGBoost and DNNs being the models with the best results. These models almost always seek to determine the occurrence or not of a specific event, which has proved to be very useful in the triage of hospitalized patients and in the search for a diagnosis.

In general, all the evaluated studies presented good results, making predictions according to the research objective. Responding to the objectives of this work, we conclude that it is possible to predict specific tests from other laboratory tests, with the complete blood count being the most used in the prediction of new results. The most used method was binary classification with supervised learning.

Thus, the use of laboratory tests and ML techniques represents an innovative potential for the process of medical laboratories, allowing for a more comprehensive analysis of the tests performed, enabling the early discovery of unknown pathologies or errors in the tests performed. This automatic analysis is very advantageous as it is low-cost and does not interfere with the processes already established by medical laboratories.

## Abbreviations

AUC: area under the curve
DNN: deep neural network
ML: machine learning
NIH: National Institutes of Health
PRISMA: Preferred Reporting Items for Systematic Reviews and Meta-Analyses
